# A momentary lack of rituals: urban festivities cancelations in Geneva, Turin, and Zurich during the COVID-19 lockdowns

**DOI:** 10.1186/s41257-023-00095-y

**Published:** 2023-09-18

**Authors:** Sandro Cattacin, Fiorenza Gamba, Nerea Viana Alzola

**Affiliations:** Institute for Sociological Research, University of Geneva, Uni Mail, 40 Bd. du Pont d’Arve, CH-1211 Geneva 4, Switzerland

**Keywords:** Rituals, Urban space, Covid-19, Memory, Belonging

## Abstract

Rituals of territorial belonging as established practices of inclusion improve dynamics of belonging and coexistence. They are particularly significant in the city where people need rituals to trust each other. But what happens when rituals disappear? The Covid-19 pandemic is an experiment on a societal level on the role of rituals. We will analyze three canceled or strongly modified traditional events in Geneva, Zurich, and Turin. We conclude that these cancellations of rituals in urban public spaces generated a lack of territorial belonging and produced mistrust and anger, but at the same time pushed people to develop new dynamics to fill this void. Frustration over the cancellation was transformed into agency. This sense of emptiness was filled through the multiplication of personal and social rituals, through the rediscovery of the city’s territory alone or with their groups of affiliation, and through new digital rituals linked to the urban ritual.

## Introduction

Analyzing rituals as fosterer of territorial belonging, that is rituals as established practices of inclusion able to improve dynamics of belonging and coexistence, is one of the three objectives of our ongoing Belgian-Swiss research project UNIC (UNexpected InClusion). The major project hypothesis states that cities are “inclusion machines” (Gamba and Cattacin 2021; Stienen et al. 2006) able to find a way for all newcomers to become part of a territory and develop feelings of belonging. Focalizing on spontaneous dynamics, migrant associations, and rituals of territorial belonging like festivals, concerts, or local sports events, we envisioned organizing our fieldwork observations during 2020 in several European cities. However, all the events we planned to observe were canceled or modified, and we could not attend the celebrations in person. As researchers, we were destabilized and forced to change or adapt our research methodology and, since March 2020, our way of living. Facing such an unexpected situation, soon, though, we realized the unique chance to verify many hypotheses hard to observe without the tragedy of the pandemic.^1^

In other words, a momentary (so we hoped) lack of rituals became a not-to-be-missed opportunity to verify, by negation, one of the main hypotheses of our project. The adapted research question became: Which alternative ways do individuals and groups utilize to fill the void left by the absence of rituals and which reactions does this absence provoke? The pandemic measures permit fieldwork on how frustration over the cancellation of rituals can be transformed into agency, leading to new dynamics and engagement in personal and social rituals. In our context of urban studies, we further question, how these reinvented rituals are linked to a territory and produce belonging.

While a large proportion of studies on the lack of rituals during the pandemic have focused either on rituals related to death, funerals or mourning, or on the appearance of invented rituals, studies on the lack of rituals related to festivals are rarer, particularly studies on the lack of urban rituals of territorial belonging and inclusion.^2^ This paper aims to be a contribution to the analysis of the latter issue.

We first introduce our conceptual framework and the topic of urban rituals as a prototype of more enduring forms of territorial belonging and, especially, focus on why such celebrations are significant for the inhabitants. Then, we present and compare the three cases and, finally, develop some research results and observations on the consequences of this momentary lack of rituals.

### Conceptual framework. Urban rituals of territorial inclusion

The interpretative key to our analysis is the territorial dimension of rituals and their absence, limitation, or transformation during the pandemic. Rituals are often analyzed as a rite of passage. Arnold van Gennep proposes to distinguish three phases of a ritual. First the separation, or pre-liminal phase during which the individual is separated from the respective community; second, the marginal or liminal phase, where the individual is in an ambiguous condition of suspension from the activities normally carried out and which represents the heart of the ritual; and third, the reintegration or post-liminal phase, in which the individual is once again admitted to the community. The achieving of these phases—in theory—normalizes moments of crisis, passages in van Gennep’s terminology, in the life of the individual, preventing society from being either harmed or damaged by them (van Gennep 1981 [1909]).

Aligned with van Gennep’s approach, rituals are considered moments of effervescence that break the established order and then reconstitute it—thus affirming the hegemonic order (Durkheim 2008 [1912]). Alternately, rituals are conceptualized as tools capable of subverting an established order. They act as an anti-structure that opposes and rebels against the structure, exercising a power of resistance or a counter-power (Hall and Jefferson 2003 [1975]).

This opposition may show itself in the form of explicit rebellion (Gluckman 1963), but more often this dynamic, defined by Victor Turner as anti-structural liminality, manifests itself as the possibility of rituals to constitute themselves as spatio-temporal areas open to thought, feeling, and will, capable of generating ways of life. In some cases these are fantasies, which acquire the power to legitimize, sometimes even on a social and political level, what initially presents itself only as hypothesis, supposition, desire, or possibility (Turner 1977).

Besides, Turner pushes his analysis further, distinguishing between two forms of interaction between individual and collective in ritual. Ritual, understood as a liminal phenomenon, consists of collective cyclical seasonal, biological, or social ritual actions expressing the experience of the community over time. Quite the reverse, ritual understood as a liminoid phenomenon, shows a production of ritual action, which does not necessarily come from a collective origin, but may well be the initiative of a single individual, who then passes it on and shares it with others—an act that produces collective effects and may develop along the margins, in the interstices, being plural, fragmentary, and often experimental (Turner 1979).

In our conceptual framework, we draw on two other dimensions of ritual. Some interpreters have stressed that rituals are effective tools to counter racism and xenophobia. What interests us here are processes of accepting otherness deployed in ritual (Augé 1994; Sennett 2012). At the same time, one of the most relevant aspects of the conceptual framework for our ritual analysis is the dynamic of formalization and informalization that some authors have focused on, such as Mary Douglas, who called these dynamics ritualism and anti-ritualism (Douglas 1996 [1970]), or Sennett (2012) who focalized on the individually differentiated intensity of the participants’ involvement.^3^

In particular, Douglas’ analysis is a fundamental starting point for understanding the transformation of rituals. Indeed, while ritualism produces highly formalized and stable practices, managed by institutions, anti-ritualism expresses a critique of rituals, their formalism, their standardized expressions, and the institutions that manage them. However, this is not a simple dichotomy, but rather a transition that leads anti-ritualism to transform itself over time into ritualism, i.e., to institutionalize. Thus, anti-ritualism draws on a whole range of reasons—relating to the alienation of certain social values—and yet all forms of rejection of rituals quickly revert to ritualism because of the need for a coherent system of expression, which is obviously necessary even for anti-ritualism (Douglas 1996 [1970]).

The *Performance Studies* approach crucially contributed to a deeper understanding of ritual practices. Indeed, it highlighted how the ritual is produced in a spatio-temporal context, endowed with specific characteristics. In this context the participants play different roles, producing through games powerful symbolic meanings (Prashizky and Remennick 2018), similar to what happens in theatre (Schechner and Schuman 1976; Turner 1982). This focus on performance, on the frame in which it takes place, invites us to insist on the role of the ritual’s space and its fundamental symbolic importance. While for Mircea Eliade a sacred space was a non-homogeneous space (Eliade 1965), i.e., completely separated from everyday space and its activities, the space of contemporary rituals, on the contrary, is a homogeneous, everyday space, which is symbolically rewritten (Pezzini 2009) during ritual practice. Because of the attribution of meaning, this space becomes the object of an affective, symbolic appropriation—an appropriation not focused on possession, but on relationships of care for a common good. These spaces are surfaces of inscription (Gamba 2009), where individuals imprint their material and symbolic traces, finding, according to Rykwert (2008), a place in the space. Rituals not only produce a bond between the participants and the place, but also foster the development of a bond, a sense of union, between the participants themselves, what we might also call a sense of community. In this sense, belonging to a place is both a weak and a primary bond (Blokland 2003). It is weak because it develops rapidly, even in newcomers. It does not require a lengthy reiteration of the practices or any specific skills or characteristics of the participants. Anyone can participate in and appropriate the ritual and the place. Also, it is primary because it can occur before any other form of bond and belonging. We have defined these rituals as territorial rituals of inclusion of differences for these reasons. The sense of belonging to a place that is produced during the ritual allows differences to be emphasized (Favole 2003) and holds together in new configurations individuals and collectivities, belonging and origins, stability and mobility, histories, and ties (Gamba et al. 2022).

The urban rituals of territorial belonging produce agency and have specific characteristics: reflexivity, indetermination, and personalization. First, the characteristic of reflexivity refers to the participants’ ability to reflect about themselves, in the sense of a capacity for self-narration and a reflexive self; to put it in Victor Turner’s words, “the ways in which a group or community seeks to portray, understand, and then act on itself” (Turner 1979). Then, indetermination indicates the possibility for the participants to have a wide margin of interpretation and multiple intensities of participation in the ritual. This condition of freedom is promising to produce a sense of belonging that spans difference. Finally, personalization is the condition that allows the individuals to participate according to their respective lifestyle, hierarchy of values, beliefs, preferences, and emotions. Accordingly, the spirit of participation in territorial urban rituals—distinctly to be distinguished from individualism—is to be with others, each in their own unique way.^4^ These three aspects characterize participation in urban rituals and foster a sense of belonging and inclusion (as we shall see in the cases analyzed), the symbolic power of which is linked to the participant’s relationship with the place. Thus, because of this link, the ritual space becomes a hyper-place (Lussault 2017), in other words, a place where symbolic practices are activated, and emotional activities take place.

## Methods

Based on different methods of data collection (interviews with stakeholders, observations, and digital ethnography) and on content and discourse analysis, we focalize on how inhabitants of cities experience the loss of urban rituals of territorial belonging. The context is the Covid-19 pandemic. We focalize on alternative practices or reconfigurations (Knoblauch and Löw 2020) which were developed during this period in response to the cancellation of urban rituals aiming to create territorial belonging. In this perspective, we take as field of analysis three major urban events situated in Geneva, Turin, and Zurich which were canceled or strongly modified during the pandemic: The Escalade in Geneva, the Celebration of San Giovanni (Saint John) in Turin, and the Sechseläuten in Zurich.

## Three case studies: The Escalade in Geneva, the San Giovanni in Turin, and the Sechseläuten in Zürich

The three chosen events are in many ways similar. They refer to an ancient narrative and are organized around a central moment at a precise hour, usually with some adjacent co-events. The three rituals are generally acknowledged as the principal, most known, and most popular event of the respective cities. They differ in the season they take place (winter, summer, and spring), and in the logic of commemoration of the traditional event (see Table 1).

**Table 1:** Some characteristics of the analyzed rituals

	Year of reference	Narrative	Main (ritual) event	Season	Duration
Escalade	17th century, regularly since 1603	- Protestant Geneva’s victory against catholic invaders in 1602	- Historical parade - Race - Religious event	Winter	7 days
San Giovanni	Middle age – 1325 Interrupted in 1853 Regularly since 1973	- Symbolic summer solstice celebration - Patron saint’s day	- Bonfire - Fireworks - Torchlight on the river	Summer	2 days
Sechseläuten	15th century, regularly since 1868	- Symbolic end of the winter - Oracle to determine the expected weather in the summer	- Burning of a snowman (dummy) on the pyre	Spring	4 days

During the first wave of the Covid-19 crisis, the three events were canceled or strongly modified. These unexpected circumstances forced us to look at these events from another angle and to investigate the meanings triggered by their absence. To this end, the next pages present the characteristics of the three rituals before the pandemic and how they were transformed during the first wave of Covid-19. In particular, we analyze how the event’s cancellation was announced by the organizers and the participants’ reactions. Then, we focus on the functional alternatives developed during this period and the emotions triggered by these changes. Finally, we explore the effects of these unexpected circumstances on the sense of belonging produced by these major events and how the organizers intend to proceed with the celebrations in the coming years.

### The Escalade in Geneva

The annual Escalade Festival commemorates Geneva’s victory over the Savoy assault on the city ramparts during the night of the 11^th^–12^th^ of December 1602. Through this attack, the Duke of Savoy’s troops intended to re-establish Catholicism in the city. However, the city of Geneva did not succumb to the Savoyard offensive and remained a Protestant city (Dufour 1997). We identified three major categories of events organized during the urban ritual: historical, religious, and festive.

- Over a weekend in mid-December, the historical parade in traditional costumes, organized by the Compagnie 1602 with volunteers, takes place. It is accompanied by several traditional songs’ performances in the public space, symbolic actions, such as for example the bonfire in the square in front of St. Pierre Cathedral, and official speeches. During the celebration Geneva households prepare the famous Escalade soup and break the chocolate cauldron, *la Marmitte, both symbolizing the legendary event when Geneva’s mayor Royaume poured a pot of boiling soup over the heads of the Savoyards* (Buscarlet and Klopmann 2017).
- The Escalade religious service proceeds after the historical parade in the Protestant St. Pierre Cathedral. While it commemorates the events of 1602, today, it also celebrates cantonal religious coexistence. Indeed, the authorities of different religions are regularly invited to attend the celebration (Schinz 2018).
- The Escalade festive events include the march of Geneva secondary school students called the *Picoulet*,^5^ the Escalade race, organized by the Race Committee with volunteers the weekend before the traditional historical parade, the competition during the race called *Marmite* during which the runners are in costume, and some neighborhood celebrations (Roth 2019).

In 2020, when the organizing committees realized that the event could not be organized safely due to the Covid-19 pandemic, the Escalade was officially canceled. On the 21st of August and then on the 1st of October, we observed a strong emotional public involvement in the official announcements and interviews made afterwards by the Race Committee (CCE 2020) and the Compagnie de 1602 (2020). The sadness and disappointment expressed over canceling such a significant event for the city were distinctly visible. However, the organizers stressed that the decision was inevitable and that the timely cancellation prevented wasting energy and economic resources in event organization (which generally lasts over a year). Above all, they emphasized that canceling event was necessary to preserve the health of all participants and volunteers. In such a feeling of care for the “Escalade’s people” we can recognize how an urban ritual, whose participants often do not know each other, can emphasize a sense of communality and cohesion (Hertz and Cattacin 2015).

Today, the public’s enthusiasm for our festivities and our parades is so strong that it is practically impossible to put in place, guarantee, and strictly enforce the measures imposed by the Federal Office of Public Health. The narrowness of the streets, sites, and esplanades of the Old Town of Geneva represent a major and obvious risk of virus spreading, which cannot be entirely controlled. We do not want to put the population and our members at risk. (Compagnie de 1602 2020)^6^

On social media, the reaction of the population to the local media’s announcement of the 2020 Escalade cancellation was controversial.

- Many comments verbalized the melancholy, nostalgia, and sense of loss in hearing this news:
  “Huge disappointment, many guests to fail, for one of the biggest and most beautiful historical processions in Europe. Exceptional previous evenings in our memories” (Léman Bleu 2020b); “What a pity!” (Tribune de Genève 2020b); “Life has become very sad with all the event cancellations” (Léman Bleu 2020b).
- Some, despite their disappointment, recognized the necessity of this decision due to the particularly complex situation:
  “Disappointed but that’s normal” (Tribune de Genève 2020a); “It’s a pity, a great disappointment, but too risky anyway: impossible barrier gestures, more than 50,000 people in the old town, crowded public transport, cafés-restaurants, stands, and sanitary facilities, etc. Impossible, therefore. Exhibition, various markets, including the Christmas market, the escalade race, etc. Yes, it’s a pity, but it takes courage.” (Léman Bleu 2020b)
- A considerable number of messages expressed understanding and support for the two organizing committees:
  “Hats off to you! President, dear Jean-Marc! Wise and responsible decision”; “Hard decision but so easy to understand. Thanks again to the whole organization for its professionalism! See you next year and in even greater numbers.” (Léman Bleu 2020a)
- However, we also observed messages of anger and mistrust that embodied adverse feelings towards Covid-19 restrictions in general:
  “The psychosis continues” (Radio Lac 2020a); “Instead people will party at home and it will be worse” (Radio Lac 2020b); “Stop canceling all events, we are like sardines on the bus and for such an important event of Geneva’s history and the bravery of its citizens... Please change your decision, it’s illogical” (Léman Bleu 2020b); “And living is still not forbidden???”; “Shame” (Léman Bleu 2020c); “Hypocrisy” (Tribune de Genève 2020a); “State decisions are abusive! How sad...” (Léman Bleu 2020b)

Only a few symbolic events took place in Geneva’s public spaces, which were not officially promoted to avoid crowding. On the day of the celebrations, for example, the president of the Compagnie 1602 placed some flower crowns in symbolic places of the city to commemorate the historic event, and the St. Pierre Cathedral had its bell ringing to the melody of “C’é qu’è lainô” every hour to remember the solemn event’s traditions. The Race Committee decided to make the event visible online by launching a special costume competition on social media (Course de l’Escalade 2020), and in the city by putting some symbols of the escalade race at several emblematic sites. For example, they placed a banner in the *Parc des Bastions* saying:

“Thank you for thinking about us on this special weekend. We miss you and we look forward to seeing you on 4^th^ and 5^th^ of December 2021.”

Several nostalgic runners photographed the banner. They retraced the traditional route in groups of friends or with their family at the time the formal event would have taken place (Ducret 2020; Léman Bleu 2020c). Indeed, even if the official events did not occur in the city’s public space, part of the traditional rituals happened in the inhabitants’ private spaces, albeit adapted. For instance, most of the time the traditional chocolate cauldron breaking took place with small *marmittes* due to crowding restrictions, and new elements were added, such as masks and hand sanitizer. Also, symbolic gestures occurred online, on local televisions, and in private companies. On the official days of the event, numerous photos and videos of the Escalade were shared on social media with the hashtags #escalade or #courseescalade to remember the moments experienced in previous years. Local and national television channels continued to broadcast new and old content about the Escalade to sustain the memory of the event. And, finally, even an outdoor escape game company decided to dedicate a special escape game to the Escalade theme (Julie 2020).

The future of the Escalade lies in the hope that people can return to celebrate the ritual as they did before. Also, the desire for the next celebration to be even more festive than in previous years is tangible in the organizers’ speeches and participants comments on social media. In 2021, the escalade race took place, and, despite the bad weather, the effervescence of participants and the public was palpable.^7^ The historical parade, however, was canceled for the second time, reproducing frustration and many of the feelings observed in 2020.

To conclude, Escalade festival’s cancellation and its transformations in 2020 produced contrasting feelings among participants. On the one hand, disappointment, nostalgia, sadness, and even anger and mistrust towards the federal and cantonal authorities were strongly voiced after the official announcements and during the days of the celebrations. On the other hand, people also expressed feelings of trust in the organizing committees, understanding and supporting the decision to postpone the festivities. Although the festival did not take place officially, emotional elements and new practices were spontaneously reproduced by people during the days of the celebrations or by the organizing committees of the Escalade. The production of memory and the use of physical and virtual space continued to offer people a complex symbolic narrative of the event. The functional equivalents were temporary substitutes for the affective narrative identity experienced during the Escalade festival in the years before (Gamba and Cattacin 2021). The lack of the urban ritual ignited the people’s need for sharing the memory of the event and even for constructing new substitute practices. Such acts demonstrate how important a ritual´s abilities to trigger identity and build a sense of belonging are. Crucially, as rituals are produced and accumulated, such sense of belonging changes and enriches experiences (Borer 2006; Gamba and Cattacin 2021). The Escalade festival appears as an urban ritual with such symbolic power for the population that when canceled, they need to re-imagine it somehow while waiting to return to the original festival performance.

### The San Giovanni in Turin

San Giovanni’s day is the celebration of the patron saint of Turin. The municipality organizes festival, with the help of several associations^8^ and private donors. The traditional celebration includes the following events:

- A bonfire on the 23^rd^ of June, *farò* in the local dialect, in which a wooden bull (*boll*), the city’s symbol, is burnt in the city center square. The city’s mayor lights the bonfire, and the direction in which the bull falls, indicates whether it will be a good or a bad year.
- Several events take place in the city center on the same day, including a historical parade with the Turin city masks Gianduja and Giacometta, organized by the Famija Turinèisa association.
- On the morning of the 24^th^, the religious ceremony happens, including the procession, the ostension of the saint’s relics during mass, and the presentation of the gift of charity to the city authorities, namely a blessed loaf of bread seasoned with pepper and saffron.
- On the evening of the same day, there is an evocative torchlight procession of boats along the River Po organized by the canoeing school Amici del fiume, attended by members of the school. The celebration ends with the most awaited moment: the fireworks show that is visible from various places in the city and beloved by the population.

In the context of Covid-19, the case of San Giovanni is specific because the adaptations due to the pandemic restrictions did not initiate ritual transformations, but merely continued—possibly accelerated—a process that had already begun 2 years beforehand in 2018, when the fireworks were removed and replaced by a drone show, for ecological, ethical, but also political reasons. The drone-show severely limited public participation even in 2019 because the projected lights were only visible from a short distance, while the fireworks could be seen from many places in the city: Piazza Vittorio, the hillside, and the River Po’s banks. That transformation drastically reduced the number of participants and raised discontent among the citizens who felt deeply deprived of a collective celebration.

Consequently in 2020, during the pandemic, the event was not really canceled but organized in a hybrid online and reduced in-presence mode, confederated with two other cities—Florence and Genoa—and transmitted by a national TV channel. Although the festival had to comply with lockdown measures, the mayor presented it as an innovative event, carried out in a financially efficient manner (400 thousand euros compared to over 700 thousand for the 2019 edition):

“We wanted to transform the limitations induced by this emergency into opportunities to enhance Turin’s cultural and artistic heritage, relying on the innovative vocation that has characterized our city for years. We are convinced that going beyond the physicality of symbolic places, changing perspectives and modes of participation, will broaden citizens’ cultural identity and sense of belonging.” (Di Paco 2020)

Thus, a year later, in the 2021 edition, citizens didn’t have a much different reaction to the further modification of the celebration—namely the streaming of the *farò* and the replacement of both drones and fireworks by a concert and an acrobatic dance show visible only via streaming. As in previous years, the most common complaint is expressed on social media by the hashtag *#ridateciifuochi* (give us back our fires). Emotions are also present, like disappointment and anger, which had already been expressed in the 2019 edition, including through official press channels:

“Every revolution demands its price. What Turin must pay for wanting to overturn San Giovanni is to have made it a festival for the few. Or at least not for the masses for the simple fact that half the people could not enjoy the spectacle: the water screen effects covered by a wall of hands and mobile phones; the drones hidden by the illuminated streetlamps. Both, too far and too few for a square over 300 meters long. And so, the innovative San Giovanni was born and died out amidst the whistles of a crowd first annoyed at the half-hour delay, then clamoring for the arrival of the sponsors as they waited to see the show begin, and finally almost silenced.” (Genta 2019)

The sarcasm common to many comments regarding alternative proposals to traditional festive events, considering their flops, can be found on various social platforms:

“great idea DJ Gabry’s concert at the Valentino, a pity that the audience couldn’t attend and that the streaming audio was bad.”^9^

Nostalgia for the traditional festival, the fireworks, but above all for being able to experience the excitement of the festival in the places where it takes place, can be read in some Facebook comments:

“Nice initiative [...] but no more streaming music [...] we need live emotions!” “Yesterday was the feast of San Giovanni, the town festival of Turin but nobody noticed”. “Yesterday afternoon around the center it seemed like a Sunday in August, [...] for some years now...San Giovanni in Turin no longer exists. It’s quite sad.”^10^

One of the torchlight procession organizers confirms the nostalgia and a feeling of loss:

Once the fires were over, the traffic was semi-blocked for an hour, indicating how many people were taking part in the day. Many times, the Torinese would dine in the restaurants near the river or in the rowing clubs, or in any case in that area, and then they would stay there or move on to see the fireworks. It was a very convivial way to end the feast of San Giovanni [...]. Let’s say that the regret and the memory of those San Giovanni are alive in many Torinese. To say how much the festival was felt, many candidates in the next municipal elections have said that they will restore the San Giovanni fires.^11^

The hope of returning to the tradition of fireworks and the places where the festivities took place in the past is a constant feeling among citizens. For the 2021 edition the municipal administration, aware of the feeling of lack produced by the radical transformation of the festival and the suppression of its strongest symbolic elements, proposed a series of alternative forms and places for the celebrations. Indeed, both the online and physical events in public space, the so-called diffused festival, were criticized. For example, the streaming events—the concert behind closed doors at the Valentino broadcasted on YouTube as well as the video projection on the walls of the Mole Antonelliana (the city’s symbolic monument) of an acrobatic dance were seen as elitist and not popular events (Redazione CronacaQui 2018; Di Paco 2021).

Other additions to the event, namely the free museums, book presentations and conferences, and the second-hand book market under the arcades in the city center, deserve a different discussion. These initiatives did not produce an emotional intensity, although participation was high in some cases, i.e., the second-hand book market of “Portici di carta”. Especially the organizing of a book market was more of a tactic that allowed inhabitants to be present together in a public space, bypassing the restrictive measures—because it was one of the few initiatives for which there was no maximum number of participants or restricted access. In a complementary way, the torchlight procession along the river, which originally ended with the start of the fireworks, was entirely deserted by the public. This shift in public presence occurred not only because of the Covid-19 restrictions but mainly because of the lack of the final event: the fireworks, a symbolic and emotional moment, the heart of the festival.

With the end of the pandemic emergency and with a change of the administration the fireworks returned in 2022. This step back was an easy way for the public administration to gain consensus on a superficial level. Still, on a deeper level, it was a way to re-establish a bond between the inhabitants and the city, which the transformation of San Giovanni had already weakened before the pandemic.

The consequences of the cancellation and transformation of the feast of San Giovanni were primarily anger and disappointment, expressed very often with sarcasm on social platforms. Added to this, there was a public distrust in the city’s public administration, which radically transformed the festival a couple of years before the pandemic. At the same time, there was a great nostalgia for the traditional form of the feast, its memory, and the construction of a narrative, at least through social media, or rather the construction of a common memory to keep the feast of San Giovanni with its fireworks alive. In addition, alongside the desire to return to the traditional form of the festival, the inhabitants expressed a strong sense of belonging to the city and its places, whose symbolic power is only partly transferred and transferable to the online dimension.

### The Sechseläuten in Zurich

The Sechseläuten (the six o’clock ringing of the church tower bells in the dialect of Zurich) is a 4-day event that celebrates the end of winter. It was celebrated since the Middle Ages but its current ritualized form originates in the second half of the nineteenth century. Five major events compose this ritual of territorial inclusion (Abgottspon 2018):

- The festivities around the guest canton invited to the event. They begin Friday at 5 p.m. and continue until Monday with stands and small events.
- The ball of the members of the guilds takes place on Saturday evening.
- Then, on Sunday children parade wearing masks. The procession has carnivalesque traits.
- On Monday afternoon there is a parade of the guilds in traditional costumes with horses and music bands.
- The burning of a snowman (a dummy called *Böögg*) laced with explosive material on the funeral pyre, usually on the third Monday in April at 6 p.m., is the central ritual. Representatives of the guilds ride around the pyre until the doll bursts. Time is measured and, according to legend, interpreted: The shorter the time, the more beautiful the summer will be.

After the cancellation of the Carnival in Basle (3 days before the event), the Sechseläuten was on the list at risk of the Swiss government decision taken the 28^th^ of February 2020 to forbid all large events. The head of communication of the organizing committee (the Central Committee of the Guilds), Victor Rosser, reacted in the local newspaper:

The fact that the Carnival in Basle is being canceled 3 days before it starts is brutal. Everything is already prepared for the big event: The magazine goes to press next week; the guests of honor and the guest canton of Uri have received their invitations. If the procession cannot take place, we would consider celebrating in the guild halls. That would be a great disappointment anyway—the last time the Sechseläuten was canceled was during the war. But we’ll have to wait and see: Until then, we remain optimistic. (Hardegger 2020)

The cancellation of the Basle Carnival and the risk of canceling the Sechseläuten shocked the organizers (“it’s brutal”, as he said). Rosser, however, remained optimistic about realizing a smaller event. Some days later, on the 6^th^ of March, the organizing committee informed the population about the possible cancellation of the whole event (Fritzsche 2020). When the Swiss government decided to reinforce the containment measures of the Covid-19 diffusion until the 19th of April 2020, they officially confirmed the event’s cancellation on the 17th of March.

The reactions were relatively modest. Comments from organizers’ side, in newspaper articles, on Facebook, or Twitter were rare. Still, three emotional elements can be identified:

- First, the feeling of sadness. For instance, a user on Twitter comments on the 6th of April that “the fact that the Sechseläuten is cancelled is pretty tough”. The organizers commented that: “The disappointment is great for all participants, guild members, guests of honor, and those involved” (Odermatt 2020).
- Second, ironic comments from people who did not like the event or felt excluded. In these comments, there was a cumulation of contestations: the youngest history of contestation of the Sechseläuten, seen as an event of a wealthy elite in Zurich (the members of the traditional guilds), as an event that does not respect animals (the horses that ride around the fire), and as a patriarchal event (only since 2014 women were admitted to march with the guilds, but until today, they do not have the right to enter in the traditional guilds).
- Third, the financial aspect was discussed, especially the fear of losing the significant amount of money already invested was publicly shared (Odermatt 2020).

Some spontaneous reactions also concerned the event itself. During the official day of the non-event, many people posted old photos of the event, memories of their participation, and historical elements about the festivity on Facebook or Twitter. They also posted burnings of small snowmen dummies at home, on the balcony, or in the neighbourhood of Letzigrund (a densely populated area), namely in the middle of a park that can be seen from many apartments. The neighbourhood’s informational journal demonstrates a series of strategies developed during the pandemic to overcome the lack of rituals. Concerning the Sechseläuten, they write:

“Even the cancellation of the Sechseläuten could be consoled at Letzigraben. Here a ‘minibögg’ was burnt—the spectators stood at the windows; keeping their distance was so easy.” (Geiser 2020)

The event was canceled twice. The announcement of the second cancellation, the 2021 edition, came in December 2020 during the second wave of the Covid-19 pandemic in Switzerland. It was a press release from the organizing committee. After having presented the technical motivation of the cancelation (impossible to guarantee security for all participants), sadness and the feeling of loss were no longer the main emotional elements present in the communication, but frustration.

“The disappointment is great, but the hope remains that there will not be another total cancelation of the Sechseläuten in the spring of 2021, but that the guilds will at least be able to celebrate in the guild parlors, of course considering the regulations then in force.” (ZZZ 2020)

In the press release and in other comments the organizer announce that they intend to assemble some smaller events. The search for functional equivalents during the pandemic begins some weeks after the announcement. The idea is to find a way to recreate an event that brings together the inhabitants of Zurich. The decision taken by the organizers is communicated on the 8th of March 2021. Together with the public television, they decided to burn the Böögg not in Zurich, but in the mountains, without a physically present audience but directly broadcast by television. The act is motivated by frustration. In the press release, the organizers write:

“After more than a year of Corona and many restrictions, the Böögg burning is also intended to provide a ray of hope for a better future. Thanks to the direct broadcast on television, all those who last year greatly missed the beautiful tradition of the Böög with the weather forecast for the summer could this year again join in the excitement and guess the burning time ahead.” (ZZZ 2021b)

Some days later, the organizers informed that they had found a place and that the ritual will take place the third Monday of April 2021 (ZZZ 2021a). The chosen location was the Schöllenenschlucht in the canton of Uri, the invited canton of the year 2020. It is a well-known national historic and symbolic place. The gorge crossed by a bridge is considered a symbol of the taming of the Alps, the conquest of the forces of nature by the technology. The reaction to the announcement was first moderated; but then, it was declared as a non-sense:

“The lack of ideas and taste of the people of Zurich obviously knows no bounds and so this nonsensical action must also be transferred to the mountain canton.”.^12^

In other comments, people were irritated about the amount of money spent on the event despite the critical pandemic period; others criticized the idea from an ecological point of view or as instrumentalization of the alps for an urban event. However, the TV station’s marketing machine and the organizers also created enthusiasm. All steps, from the production of the Böögg, to the transport of the Böögg to the alps, the construction of the pyre, with all the difficulties, and finally the main event with the burning of the snowman could be followed on social media, in newspapers, and on (both local and national) television channels. And after the event, comments about all details—and clearly the time the burning lasted—were discussed in all contexts, even in the city that opened the outdoor terraces of the restaurants the same day (the 19th of April) (Arnet 2021).

The consequences of the absence of the Sechseläuten, which we summarized shortly, can be read in its development in four periods. First, after the announcement of the event’s cancelation, the feeling of loss was omnipresent, and sadness was the main reaction. Many inhabitants of Zurich tried to reproduce the ritual at home or in semi-public spaces. During this moment of loss, many narratives of the past came up, like remembering the loss of a beloved. When the second cancelation was unavoidable, the loss was transformed into frustration, shared through social media and the organizers’ press releases. This frustration ignited the reinvention of the ritual, the creation of a new form of public event. It was transmitted only in the TV but followed by the public like a ritual with all the elements necessary to create emotions and expectations. All the steps of the event were explained and commented in the days after. The reimaginations of the festivity can also be analysed from the point of view of the loss of a public space. It was first privatized, then shared to create a common experience, and finally transformed into a utopian alternative, in the mountains, in a space that belongs to nobody and everybody. This hybrid experience could find a place in the future narrative of the ritual. It was able to cancel the feeling of loss and combine the event with signs of resistance to the pandemic: it has taken place against all odds, it was in the alps, at a place that represent the strength of the society against the nature. The coexistence of man and nature, a theme included in the Sechseläuten ritual, was thus confirmed and continued through this hybrid performance.

## Discussion

What happens when urban rituals cannot occur as usual because of an unexpected situation like the Covid-19 pandemic? In the three cases analysed, we noted that the announcement of the event cancelation or modification triggered feelings of sadness and nostalgia. Functional equivalents were produced from the organizers’ side and spontaneously by inhabitants of the cities in public and private spaces. Social media and forms of broadcasting also filled the momentary lack of rituals. Finally, we observed that (critical) discussions about the event’s future took place on several occasions and in different forms on social media and public speeches, especially in Turin and Zurich. These debates were fueled by discontent among the inhabitants and could contribute to a partial renewal of the event (see Table 2).

**Table 2:** Descriptive comparison of the lack of urban ritual of territorial belonging

	Form of the announcement	Functional alternatives during the cancelation	Plans for the future of the ritual
Escalade	Loss, economic consequences, protecting health	- Commemorative flower crowns at symbolic sites - Symbolic races (spontaneous) - Cathedral bell - Event visibility (online and in the city) - New ritual objects (masks and hand sanitizer)	Returning to the traditional event
San Giovanni	Loss and anger already before the pandemic	- Small events in the city, hybrid events	Contested development of an event in difficulties, hope of a return to the form changed before the pandemic
Sechseläuten	Loss, economic consequences, then frustration, then reflexivity and agency	- Privatization, small private and associative bottom-up events - Reinvention of the ritual as a hybrid event	Returning to the traditional event, confrontation with critics

A common feature in this dynamic of *filling the void* was remembering previous years’ festivities and historical moments. For example, in Zurich, we observed circulating videos and photos of a Sechseläuten event that took place without horses because they were sick, or the inhabitants remembered that only once the celebration was canceled during the war. Similarly, in the case of Geneva, there was nostalgic photo sharing on social media and historical reconstruction of the few exceptional years when the event had not been celebrated. In Turin, though, besides nostalgia for past celebrations, sharing photos and videos on social media emphasized the feeling of anger towards the event organizers, which marks a clear distance between the latter and the participants. In any way, these digital acts of remembrance indicate that in all three cases—in contrast to the ritual performances—the work on the narrative was not interrupted. The online narratives concerning memory, nostalgia, but also sadness or contestation were able to keep alive a sense of community and belonging in a condition of lack of rituals.

In filling the void, we can find, contextualized to the pandemic situation, the dynamic described by Mary Douglas between ritualism and anti-ritualism. It was indeed a unique context that offered the possibility of examining the transformation of anti-ritualism into ritualism. For example, the pleas not only to maintain rituals, but more the hope and desire to still be able to celebrate rituals in their stable and institutionalized (traditional) forms are common to the three cases analyzed in this paper. It should also be added that, in a complementary manner, the spontaneous forms of ritualization that appeared during Covid-19, either in the form of substituted rituals, mostly online, or in the form of the invented rituals, such as clapping from balconies, represent fast forms of ritualization. As such, the unique situations of the pandemic reinforced theories that recognize rituals as having a fundamental role in formulating the basic needs of social interaction, emotional support, and sense of continuity.

The strong need for rituals, explicated in multiple ways, including through narratives delivered in digital spaces, exemplifies how the Covid-19 pandemic and the ensuing lockdown characterized a spatio-temporal liminality that brings us back to Turner’s analysis. Between the separation from the pre-Covid-19 life and the expectation of re-integrating a post-Covid-19 life, whose caesura from the previous one was already foreshadowed, the lockdown was an example of an anti-structural liminality in which uncertainty, instability, reconfiguration of hierarchies, power dynamics, and social roles did not prevent individuals from generating ways of life open to thought, feeling, will, and imagination, appropriating all kinds of available spatio-temporal contexts, very often digital, more rarely physical.

The three cases analyzed in this paper show that the search to keep rituals alive through a collective narrative or alternative forms was the way to fill a void, firmly considered and desired as temporary. Ritualizations presented a way to make sense of and make endurable an extremely long and exhausting liminality. Moreover, this need to participate in rituals, makes it extremely clear how during the pandemic, precisely through even minimized forms of rituals, those generalized social bonds where developed through the experience of equality in liminality, which Turner calls communitas, without which there could no be a society based on respect (Turner 1969: 96). A bond that we have also seen emerge in the ritual of applause from balconies and which shows, once again, how one does not live without rituals (Gamba 2020).

Although the rituals have been affected by the pandemic, their affective narratives persist in the sense of a territorial belonging transformed—reinterpreted or invented—through a process of imagination (Anderson 2006 [1983]). On the organizational level, social media and broadcasting were used to create hybrid ritual moments of communality, appreciated, and received differently in the respective cases. However, the *online transfer of rituals* has raised, in addition to its considerable advantages, some shortcomings: besides multiplying, extending, bringing closer, in other words hybridizing, physical spaces, people and relationships, the digital alternatives were not able to fill the void, or replace the physical presence of participants in a ritual, as many comments underlined (Iranmanesh and Alpar Atun 2020) (see also Table 3).

**Table 3:** Consequences of the absence of urban ritual of territorial belonging

	Major reactions of the population on the announcement	Major reactions of the population during the days the event is canceled	Major reactions of the population on the proposed alternatives
Escalade	- Sadness, Loss - Understanding - Anger - Mistrust	- Nostalgia (online) - Symbolic actions (Public space) - Family celebrations (Private)	- Disappointment - Support - Spontaneous actions (bottom-up)
San Giovanni	- Loss - Mistrust - Remembering activities - Sarcasm	- Substitute activities (public space)^a^ - Illegal initiatives - Feeling of exclusion	- Critics - Political distrust - Love for the City
Sechseläuten	- Loss - Remembering activities in the social media during the days of the event	- Privatization of the event - Semi-public events	- Criticism first, then inclusion of the hybrid event in the narrative of the ritual

^a^Already in the years before the pandemic

The main differences can be seen in the fact that the Escalade and the Sechseläuten are also based on a ritual object that can be easily reproduced (the Snowman^13^) or bought (the chocolate cauldron). On the contrary, San Giovanni does not involve individual and easily reproducible ritual objects: its symbols are collective and need a public space, the bull burnt in the *faro* (that has no distinctive element that can be used in an equivalent way), the fireworks (but the same goes for the drone show), the torchlight procession along the Po River. In other words, in the case of San Giovanni there are no objects that can replace the symbolic meaning of the performances. Probably because of the prior contestations, the organizers did not “use” symbolism that could have been invented (for example the masks) such as the Zurich organizers did with a paper cut Snowmen to burn. Hence, the public focus and debate was already set before the pandemic, and thus also limiting the field of creativity within the reimaginations of this contested ritual.

In Geneva and Zurich, the personalization of the ritual became possible thanks to this ritual object. But, in contrast to previous years, such individual ritualistic acts were communalized by social media. Sharing of the private (often ironic) moment with others created a hybrid space of belonging to a real territory. The Sechseläuten in Zurich shows, as while the formalized re-imagined version was controversial, the informalization of a very elitist ritual (by burning your own Snowman in the neighborhood) kept the narrative going just as much as the formal organized version and thus it opened a space for constructive anti-ritualism and alternative community and belonging creating acts.

## Conclusions

During the Covid-19 pandemic, the urban rituals and festive activities in Geneva, Zurich, and Turin were canceled or strongly modified. These cancelations of ritual performances in urban public spaces, or the shift in their symbolic locations, generated a lack of territorial belonging. When the physical space of urban rituals became no longer accessible due to restrictions, it created a sense of emptiness. These circumstances produced mistrust, anger, but at the same time pushed people to develop new dynamics to fill this void.^14^ Frustration over the cancelation was transformed into agency. This sense of emptiness was filled through the multiplication of personal and social rituals, through the rediscovery of the city’s territory alone or with their groups of affiliation (jogging, parks, etc.), and through new digital rituals linked to the urban ritual. The pandemic made it impossible for urban rituals to take place in a public space where it was possible to establish such a special bond between place and participants.

Therefore, the digital technologies and the most widespread social networks, like Facebook, Twitter, YouTube, and Instagram, played an irreplaceable role in the different spheres of social and individual life, not only to continue vital or routine activities, but also to maintain ties and the sense of belonging of individuals to a community and to a place. This has been revealed in a tragic way with the rituals related to death and mourning (Pentaris 2021), but urban rituals of territorial belonging, such as those presented in this article, have also made use, in a more or less intentional and organized manner, of various digital devices.

The organizers and the public draw on variations of hybridization to re-imagine the territorial rituals. The link between the physical and spatial context of a ritual and the online interaction connected to it is certainly not an effect of the pandemic, and the creation of hybrid event communities resulting from the combination of online and offline ritual practices has already been analyzed (Simons 2019). Similarly, in the era of the *network society* (Castells 1996) and of *personalized networking* (Wellman 2001) online and offline are not separate, but constitute the environment, the *onlife* (Floridi 2015), in which individuals act. Thus, individuals reconstitute urban rituals in this space, connecting people and place over networks.

The narratives of the events were kept alive by the people and the organizers of the festivities in several ways. We observed that the places of these rituals changed, some symbolic activities had undergone transformations. However, the collective and individual narratives linked to the memory of the festivities ensured a sense of continuity. Memory maintained a bond among people, between them and space, in its physical and digital configurations. Memory was reproduced through collective and individual narratives and tied to the places and their identities. These narratives also changed during the pandemic—and will probably continue to transform in the future—but they represented that common thread that ensured continuity, meaning, and belonging during this time of uncertainty. On a symbolic level, indeed, urban rituals continued to manifest how the city represents an inscription surface on which people leave their traces (Gamba 2009). However, to become a hyper-place where symbolic practices and emotions are activated, urban rituals need to be performed in the places where memory resides. Indeed, on a material level, people’s desire to return to performing ritual in public space has been strongly expressed. Rituals in private or digital spaces seem to have filled the void only temporarily.

Our analysis thus shows that—beyond such exceptional moments of crisis—the material and symbolic dimensions together make it possible to create a ritual of territorial belonging (Lowe 2000). The ritual of territorial belonging needs to be performed through the body in a physical space where we perceive “the existential and phenomenological reality of place” (Low 2003), where memory resides, and other people participate. However, this combination of elements is not possible in the digital space; that is why the observed digital rituals seem to fill the “void” only temporarily.^15^ Interestingly, however, the pandemic has also accelerated the creation of rituals of territorial belonging in their hybrid form (de Souza e Silva 2016), digital and physical—an object of study that we believe deserves more attention in future research.

## Data Availability

The research data is stocked on the University of Geneva secure drive (Switch) and can be consulted (by addressing a demand to the authors).

## Footnotes

1 The void was filled by many other initiatives at our faculty; in particular, our team produced probably the first book analyzing the pandemic from a social science point of view (Gamba et al. 2020).

2 Examples of studies for digital rituals of mourning are discussed in Asgari et al. (2022); a first analysis of the invention of rituals during the Covid-19 period can be find in Imber-Black (2020).

3 See also Abrahams (1973).

4 Referring to Derrida, Ravinder Sidhu and Donata Rossi-Sackey speak about affective hospitality that creates belonging through emotions (Sidhu and Rossi-Sackey 2022).

5 The *Picoulet* is a widespread practice in French-speaking Switzerland of forming a circle to sing and dance.

6 We translated all the texts mentioned in the article.

7 We attended the event and made some field observations.

8 Like the Famija Turinèisa, Amici del fiume, etc.

9 Comment on YouTube on the announcement of Gabry’s presence instead of the San Giovanni feast.

10 Comment on the tourism office’s facebook site “Torino da scoprire”.

11 Citation from an interview with an organizer (21.06.2021).

12 Online comment in the popular newspaper *Blick* (14.04.2021).

13 For instance, the organizer in Zurich produced a paper model to download from their site and to transform into a small snowman dummy.

14 As Tali Hatuka points out in his article on social movements during the pandemic, innovation and creativity were one result of the difficulties to organize protest (Hatuka 2022).

15 As also evidenced in the case analyzed by Iranmanesh and Alpar Atun (2020).

## References

[CR1] Abgottspon Elisabeth (2018). Sechseläuten.

[CR2] Abrahams, Roger. 1973. Ritual for fun and project. In *Burg-Wartenstein conference, no. 59: Ritual and reconciliation, New York, July 21–29, 1973*.

[CR3] Anderson, Benedict. 2006 [1983]. *Imagined communities: Reflections on the origin and spread of nationalism*. London: Verso Books.

[CR4] Arnet, Helene. 2021. Der Zürcher Böögg schickte Corona nach 13 Minuten zum Teufel. *Tages-Anzeiger*, 19.04.2021.

[CR5] Asgari Zahra, Naghavi Azam, Abedi Mohammad Reza (2022). Death and dying in a digitalized world. OMEGA - Journal of Death and Dying.

[CR6] Augé Marc (1994). Pour une anthropologie des mondes contemporains.

[CR7] Blokland Talja (2003). Urban bonds.

[CR8] Borer Michael Ian (2006). The location of culture: The urban culturalist perspective. City & Community.

[CR9] Buscarlet Luc, Klopmann André (2017). La nuit des longues échelles et autres événements du mois de décembre à Genève.

[CR10] Castells Manuel (1996). The rise of the network society.

[CR11] CCE, Comité de la Course de L’Escalade. 2020. *La Course de l’Escalade choisit la voie de la sécurité et reporte la 43e édition en 2021*. [Press release].

[CR12] Compagnie de 1602. 2020. *La Compagnie de 1602 renonce aux festivités de l’Escalade 2020*. [Press release].

[CR13] Course de l’Escalade. 2020. Déguisement de la Course de la Marmite. Facebook.

[CR14] de Souza e Silva Adriana (2016). From cyber to hybrid. Space and Culture.

[CR15] Di Paco, Leonardo. 2020. Torino, sarà una festa di San Giovanni al cubo: E lo spettacolo dei droni lo faranno i francesi. *La Stampa*, 5 Giugno 2020.

[CR16] Di Paco, Leonardo. 2021. San Giovanni a Torino: Quest’anno addio ai droni ma niente fuochi. *La Stampa*, 21 Aprile 2021.

[CR17] Douglas, Mary. 1996 [1970]. *Natural symbols. Explorations in cosmology*. London: Routledge.

[CR18] Ducret, Benoît. 2020. Course de l’Escalade 2020 Facebook.

[CR19] Dufour Alfred (1997). Histoire de Genève.

[CR20] Durkheim, Emile. 2008 [1912]. *Les formes élémentaires de la vie religieuse*. Paris: PUF.

[CR21] Eliade Mircea (1965). Le sacré et le profane.

[CR22] Favole Adriano (2003). Resti d’umanità. Vita sociale del corpo dopo la morte.

[CR23] Floridi Luciano (2015). The onlife manifesto: Being human in a hyperconnected era.

[CR24] Fritzsche, Daniel. 2020. Das Sechseläuten fällt dem Coronavirus zum Opfer. *Neue Zürcher Zeitung*, 13.03.2020.

[CR25] Gamba Fiorenza (2009). Leggere la città: Indizi di contaminazioni sociologiche.

[CR26] Gamba Fiorenza, Gamba Fiorenza, Nardone Marco, Ricciardi Toni, Cattacin Sandro (2020). On ne (sur)vit pas sans rituels. COVID-19. Le regard des sciences sociales.

[CR27] Gamba Fiorenza, Cattacin Sandro (2021). Urbans rituals as spaces of memory and belonging: A Geneva case study. City, Culture and Society.

[CR28] Gamba Fiorenza, Nardone Marco, Ricciardi Toni, Cattacin Sandro (2020). COVID-19. Le regard des sciences sociales.

[CR29] Gamba Fiorenza, Cattacin Sandro, Bob White W (2022). Créer la ville. Rituels territorialisés d’inclusion des différences.

[CR30] Geiser, Barbara. 2020. Ein Virus legt still – und bewegt. *sge Kultur*, (Oktober): 8–11.

[CR31] Genta, Federico. 2019. Festa di San Giovanni a Torino: la “Night Experience” sarà riservata a 38 mila persone. *La Stampa*, 21 Giugno 2019.

[CR32] Gluckman Max (1963). Order and rebellion in tribal Africa.

[CR33] Hall, Stuart, and Tony Jefferson. 2003 [1975]. *Resistance through rituals: Youth subcultures in post-war Britain*. London: Routledge.

[CR34] Hardegger, Angelika. 2020. Coronavirus: Welche Veranstaltungen werden nun abgesagt? *Neue Zürcher Zeitung*, 28.02.2020.

[CR35] Hatuka Tali (2022). Public space and public rituals: Engagement and protest in the digital age. Urban Studies.

[CR36] Hertz, Ellen, and Sandro Cattacin. 2015. Traditions vivantes dans l’espace urbain: une provocation? In *Lebendige Traditionen in der urbanen Gesellschaft*, ed. Bundesamt für Kultur and Schw. Akademie der Geistes- und Sozialwissenschaft, 33–40. Baden-Dättwil: Hier und Jetzt.

[CR37] Imber-Black Evan (2020). Rituals in the time of COVID-19: Imagination, responsiveness, and the human spirit. Family Process.

[CR38] Iranmanesh Aminreza, AlparAtun Resmiye (2020). Restricted spatiality and the inflation of digital space, an urban perspective. Space and Culture.

[CR39] Julie. 2020. Ah, la drôle d’Escalade! *Tribune de Genève*, 2020.

[CR40] Knoblauch Hubert, Löw Martina, Volkmer Michael, Werner Karin (2020). Die Refiguration von Räumen in Zeiten der Pandemie. Die Corona-Gesellschaft. Analysen zur Lage und Perspektiven für die Zukunft.

[CR41] Léman bleu. 2020a. Interview à Jean-Marc Barberis de la Compagnie 1602. Facebook.

[CR42] Léman bleu. 2020b. Le cortège de l’Escalade est annulé. Facebook.

[CR43] Léman bleu. 2020c. Rendre hommage à la Course de l’Escalade. Facebook.

[CR44] Low Setha M (2003). Embodied space (s) anthropological theories of body, space, and culture. Space and Culture.

[CR45] Lowe Seana S (2000). Creating community: Art for community development. Journal of Contemporary Ethnography.

[CR46] Lussault Michel (2017). Hyper-lieux. Les nouvelles géographies de la mondialisation.

[CR47] Odermatt, Zéline. 2020. Urner Auftritt am Sechseläuten ist auf 2021 verschoben. *Luzerner Nachrichten*, 17.03.2020.

[CR48] Pentaris Panagiotis (2021). Death, grief and loss in the context of COVID-19.

[CR49] Pezzini Isabella (2009). Roma: Luoghi del consumo, consumo dei luoghi.

[CR50] Prashizky Anna, Remennick Larissa (2018). Celebrating memory and belonging: Young Russian Israelis claim their unique place in Tel-Aviv’s urban space. Journal of Contemporary Ethnography.

[CR51] Radio Lac. 2020a. La Course de l’Escalade n’aura pas lieu. Facebook.

[CR52] Radio Lac. 2020b. Le Cortège historique de l’Escalade annulé. Facebook.

[CR53] Redazione CronacaQui. 2018. Festa di San Giovanni, dopo lo show dei droni arrivano i fuochi d’artificio. Indaga la polizia. *CronacaQui*, 25 Giugno 2018.

[CR54] Roth Henri (2019). Les mascarades oubliées de l’Escalade. L’envers du décor de la fête patriotique genevoise.

[CR55] Rykwert Joseph (2008). La seduzione del luogo.

[CR56] Schechner Richard, Schuman Mady (1976). Ritual, play, and performance: Readings in the social sciences/theatre.

[CR57] Schinz Olivier (2018). Escalade.

[CR58] Sennett Richard (2012). Together: The rituals, pleasures and politics of cooperation.

[CR59] Sidhu Ravinder, Rossi-Sackey Donata (2022). Creating hospitable urban spaces: A multicultural city for refugees and asylum seekers. Urban Studies.

[CR60] Simons Ilja (2019). Events and online interaction: The construction of hybrid event communities. Leisure Studies.

[CR61] Stienen Angela, Widmer Jacqueline Truffer, Blumer Daniel, Tschannen Pia, Ammann Eva Soom (2006). Integrationsmaschine Stadt?: Interkulturelle Beziehungsdynamiken am Beispiel von Bern.

[CR62] Tribune de Genève. 2020a. Course de l’Escalade repoussée. Facebook.

[CR63] Tribune de Genève. 2020b. La Compagnie 1602 annule tout. Facebook.

[CR64] Turner, Victor. 1969. *The Ritual Process: Structure and Antistructure*. New York: PAJ Publications.

[CR65] Turner Victor (1977). Process, system, and symbol: A new anthropological synthesis. Daedalus.

[CR66] Turner Victor (1979). Frame, flow and reflection: Ritual and drama as public liminality. Japanese Journal of Religious Studies.

[CR67] Turner Victor (1982). From ritual to theatre. The human seriousness of play.

[CR68] van Gennep, Arnold. 1981 [1909]. *Rites de passages*. Paris: Dunod.

[CR69] Wellman Barry (2001). Physical place and cyberplace: The rise of personalized networking. International Journal of Urban and Regional Research.

[CR70] ZZZ, Zentralkomitee der ZünfteZürichs (2020). Kein Volksfest am Sechseläuten 2021.

[CR71] ZZZ, Zentralkomitee der ZünfteZürichs (2021). Sechseläuten 2021: Der Böögg verbrennt ausnahmsweise in der Schöllenenschlucht im Kanton Uri!.

[CR72] ZZZ, Zentralkomitee der ZünfteZürichs (2021). Sechseläuten 2021: Nur der Böögg darf Feuer fangen.

